# Predictors and Outcomes of Excellent Recanalization Versus Successful Recanalization After Thrombectomy in Proximal and Distal Medium Vessel Occlusion Strokes: A Multinational Study

**DOI:** 10.1161/SVIN.124.001421

**Published:** 2024-10-25

**Authors:** Vivek Yedavalli, Hamza Adel Salim, Basel Musmar, Nimer Adeeb, Kareem El Naamani, Nils Henninger, Sri Hari Sundararajan, Anna Luisa Kühn, Jane Khalife, Sherief Ghozy, Luca Scarcia, Benjamin Y. Q. Tan, Jeremy J. Heit, Robert W. Regenhardt, Nicole M. Cancelliere, Joshua D. Bernstock, Aymeric Rouchaud, Jens Fiehler, Sunil Sheth, Muhammed Amir Essibayi, Ajit S. Puri, Christian Dyzmann, Marco Colasurdo, Xavier Barreau, Leonardo Renieri, João Pedro Filipe, Pablo Harker, Răzvan Alexandru Radu, Mohamad Abdalkader, Piers Klein, Thomas R. Marotta, Julian Spears, Takahiro Ota, Ashkan Mowla, Pascal Jabbour, Arundhati Biswas, Frédéric Clarençon, James E. Siegler, Thanh N. Nguyen, Ricardo Varela, Amanda Baker, David Altschul, Nestor R. Gonzalez, Markus A. Möhlenbruch, Vincent Costalat, Benjamin Gory, Christian Paul Stracke, Mohammad Ali Aziz‐Sultan, Constantin Hecker, Hamza Shaikh, David S. Liebeskind, Alessandro Pedicelli, Andrea M. Alexandre, Illario Tancredi, Tobias D. Faizy, Erwah Kalsoum, Boris Lubicz, Aman B. Patel, Vitor Mendes Pereira, Adrien Guenego, Adam A. Dmytriw, the MAD MT Investigators

**Affiliations:** ^1^ Department of Radiology Division of Neuroradiology Johns Hopkins Medical Center Baltimore MA; ^2^ Neuroendovascular Program Massachusetts General Hospital & Brigham and Women's Hospital Harvard Medical School Boston MA; ^3^ Department of Neurosurgery and Interventional Neuroradiology Louisiana State University LA; ^4^ Department of Neurosurgery Thomas Jefferson University Philadelphia PA; ^5^ Department of Neurology University of Massachusetts Chan Medical School Worcester MA; ^6^ Department of Endovascular Neurosurgery and Neuroradiology NJMS Newark NJ; ^7^ Division of Neurointerventional Radiology Department of Radiology University of Massachusetts Medical Center Worcester MA; ^8^ Cooper Neurological Institute Cooper University Hospital Cooper Medical School of Rowen University Camden NJ; ^9^ Departments of Neurological Surgery & Radiology Mayo Clinic Rochester MN; ^10^ Department of Neuroradiology Henri Mondor Hospital Creteil France; ^11^ Department of Medicine Yong Loo Lin School of Medicine National University of Singapore Singapore Singapore; ^12^ Division of Neurology Department of Medicine National University Hospital Singapore Singapore; ^13^ Department of Interventional Neuroradiology Stanford Medical Center Palo Alto CA; ^14^ Neurovascular Centre Divisions of Therapeutic Neuroradiology and Neurosurgery St. Michael Hospital University of Toronto Toronto ON Canada; ^15^ Department of Neurosurgery Brigham and Women's Hospital Harvard Medical School Boston MA; ^16^ Neuroradiology Department University Hospital of Limoges Dupuytren Université de Limoges XLIM CNRS UMR Limoges France; ^17^ Department of Diagnostic and Interventional Neuroradiology University Medical Center Hamburg‐Eppendorf Hamburg Germany; ^18^ Department of Neurology UTHealth McGovern Medical School Houston TX; ^19^ Department of Neurological Surgery and Montefiore‐Einstein Cerebrovascular Research Lab Montefiore Medical Center Albert Einstein College of Medicine Bronx NY; ^20^ Neuroradiology Department Sana Kliniken Lübeck GmbH Lübeck Germany; ^21^ Department of Interventional Radiology Oregon Health and Science University Portland OR; ^22^ Interventional Neuroradiology Department Bordeaux University Hospital Bordeaux France; ^23^ Interventistica Neurovascolare Ospedale Careggi di Firenze Florence Italy; ^24^ Department of Diagnostic and Interventional Neuroradiology Centro Hospitalar Universitário do Porto Porto Portugal; ^25^ Department of Neurology University of Cincinnati Medical Center Cincinnati OH; ^26^ Department of Neuroradiology Gui de Chauliac Hospital Montpellier University Medical Center Montpellier France; ^27^ Departments of Radiology & Neurology Boston Medical Center Boston MA; ^28^ Department of Neurosurgery Tokyo Metropolitan Tama Medical Center Tokyo Japan; ^29^ Division of Stroke and Endovascular Neurosurgery Department of Neurological Surgery Keck School of Medicine University of Southern California (USC) Los Angeles CA; ^30^ Department of Neurosurgery Westchester Medical Center at New York Medical College Valhalla NY; ^31^ Department of Neuroradiology Pitié‐Salpêtrière Hospital Paris France; ^32^ GRC BioFast Sorbonne University Paris France; ^33^ Department of Neurology Centro Hospitalar Universitário do Porto Porto Portugal; ^34^ Department of Neurosurgery Cedars‐Sinai Medical Center Los Angeles CA; ^35^ Sektion Vaskuläre und Interventionelle Neuroradiologie Universitätsklinikum Heidelberg Heidelberg Germany; ^36^ Department of Interventional Neuroradiology Nancy University Hospital Nancy France; ^37^ INSERM U1254, IADI Université de Lorraine Vandoeuvre‐les‐Nancy France; ^38^ Department of Radiology Interventional Neuroradiology Section University Medical Center Münster Münster Germany; ^39^ Departments of Neurology & Neurosurgery Christian Doppler Clinic Paracelsus Medical University Salzburg Salzburg Austria; ^40^ UCLA Stroke Center and Department of Neurology Department UCLA Los Angeles CA; ^41^ UOSA Neuroradiologia Interventistica Fondazione Policlinico Universitario A. Gemelli IRCCS Roma Rome Italy; ^42^ Department of Neurology Hôpital Civil Marie Curie Charleroi Belgium; ^43^ Department of Radiology Neuroendovascular Program University Medical Center Münster Germany; ^44^ Department of Diagnostic and Interventional Neuroradiology Erasme University Hospital Brussels Belgium

**Keywords:** acute ischemic stroke, mechanical thrombectomy, medium vessel occlusions, mTICI score

## Abstract

**Background:**

Acute ischemic stroke arising from medium vessel occlusions (MeVO) poses substantial challenges in treatment and management. This study aims to elucidate the outcomes and factors contributing to achieving excellent recanalization (modified Thrombolysis in Cerebral Infarction [mTICI] 2c–3) versus successful recanalization (mTICI 2b) in patients with MeVO stroke undergoing mechanical thrombectomy (MT).

**Methods:**

We conducted a multinational study analyzing data from the MAD‐MT (Multicenter Analysis of Distal Medium Vessel Occlusions: Effect of Mechanical Thrombectomy) registry, encompassing 37 centers across North America, Asia, and Europe, collected between September 2017 and July 2023. The study included acute ischemic stroke patients with MeVO treated with MT, with or without intravenous thrombolysis, who achieved mTICI 2b–3 post‐MT.

**Results:**

Among 1463 patients with successful recanalization (mTICI 2b–3), 523 achieved mTICI 2b recanalization, and 940 achieved mTICI 2c–3. Distal occlusions exhibited higher odds of excellent recanalization compared with proximal MeVO vessel occlusions (odds ratio, 1.58; 95% CI, 1.17–2.15; *P* = 0.003). Cardioembolic stroke pathogenesis was associated with a higher likelihood of excellent recanalization (1.67; 95% CI, 1.07–2.59; *P* = 0.018). Patients achieving mTICI 2c–3 recanalization demonstrated lower initial National Institutes of Health Stroke Scale scores, significant improvements in postprocedural National Institutes of Health Stroke Scale shift, and a higher percentage of favorable 90‐day outcomes compared with those with mTICI 2b. However, no significant difference in 90‐day mortality rates was observed.

**Conclusion:**

This study underscores that among patients with MeVO stroke with successful recanalization (mTICI 2b–3) there is higher likelihood of achieving excellent recanalization (mTICI 2c–3) in distal occlusions and cardioembolic pathogenesis. mTICI 2c–3 scores post‐MT correlate with improved clinical outcomes compared with mTICI 2b, affirming the superiority of excellent recanalization over successful recanalization in patients with MeVO stroke. Further prospective studies and randomized controlled trials are warranted for validation.

## Graphical abstract

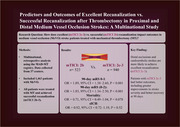

**Nonstandard Abbreviations and Acronyms**
AIS: acute ischemic stroke
IVT: intravenous thrombolysis
MeVO: medium vessel occlusion
MT: mechanical thrombectomy
mTICI: modified Thrombolysis in Cerebral Infarction
NIHSS: National Institutes of Health Stroke Scale

**Clinical Perspective**

**What Is New?**

- This study demonstrates that distal medium vessel occlusionsand cardioembolic strokes are more likely to achieve excellent recanalization (modified Thrombolysis in Cerebral Infarction 2c–3) following mechanical thrombectomy , compared with proximal occlusions and other stroke pathogeneses.

**What Are the Clinical Implications?**

- Achieving excellent recanalization (modified Thrombolysis in Cerebral Infarction 2c–3) is associated with better functional outcomes, such as improved National Institutes of Health Stroke Scale shifts and higher rates of favorable 90‐day modified Rankin Scale scores, compared with modified Thrombolysis Cerebral Infarction 2b recanalization.

**Clinical Relevance**

- Clinicians should consider these findings when treating patients with medium vessel occlusions, as achieving mTICI 2c–3 is crucial for improving poststroke recovery and functional independence.

Acute ischemic stroke (AIS), a leading cause of morbidity and mortality globally, presents a significant healthcare challenge. Medium vessel occlusions (MeVO) are particularly noteworthy, constituting approximately 25%–40% of AIS cases.^1^, ^2^, ^3^ Traditionally, intravenous thrombolysis (IVT) has been the primary treatment modality for AIS resulting from MeVO.^2^, ^4^ However, the evolving landscape of stroke management has witnessed a paradigm shift towards mechanical thrombectomy (MT), with current research focusing on its efficacy in select MeVO cases.^2^, ^5^, ^6^, ^7^, ^8^, ^9^, ^10^, ^11^, ^12^, ^13^, ^14^, ^15^, ^16^

A crucial determinant of MT success in AIS irrespective of the total number of passes is the degree of recanalization, typically evaluated using the modified Thrombolysis in Cerebral Infarction (mTICI) scoring system.^17^, ^18^, ^19^ Successful recanalization, defined as mTICI scores of 2b or higher, is linked to improved patient outcomes. Notably, the extent of recanalization in patients who attain successful outcomes, particularly those achieving mTICI scores of 2c and 3 (excellent recanalization), has been correlated with early neurological improvement and superior clinical outcomes compared t those reaching mTICI 2b.^20^, ^21^, ^22^, ^23^, ^24^, ^25^, ^26^, ^27^, ^28^

Given this background, our study aimed to identify factors independently associated with mTICI 2c–3 recanalization in patients with AIS resulting from MeVO who have achieved successful recanalization (mTICI 2b–3) and to compare the safety and functional outcomes of excellent recanalization over successful recanalization.

## Methods

The data supporting our findings are available from the corresponding author and after approval from the MAD‐MT consortium upon reasonable request. This investigation was part of an analysis of the MAD‐MT (Multicenter Analysis of Primary Distal Medium Vessel Occlusions: Effect of Mechanical Thrombectomy) registry.^5^, ^29^, ^30^, ^31^, ^32^, ^33^, ^34^, ^35^, ^36^, ^37^, ^38^, ^39^ The study received approval from the institutional review board or local ethical standards committee at each participating site, and informed consent from patients was waived. The data supporting this study's findings are available from the corresponding author upon reasonable request. This study is reported according to the Strengthening the Reporting of Observational Studies in Epidemiology guideline.^40^, ^41^

### Study Population

Inclusion criteria for this analysis were as follows: (1) patients with AIS due to MeVO; (2) MT with or without IVT; and (3) available mTICI data after MT. Exclusion criteria encompassed: (1) unsuccessful recanalization (mTICI 0–2a); (2) treatment with intra‐arterial thrombolysis; and (3) stroke due to malignancy and rheumatological diseases (eg, systemic lupus erythematosus, vasculitis).

### Setting and Ethical Approval

Characteristics and outcomes of consecutive patients with AIS due to primary medium‐proximal vessel occlusion (M2, A1, P1) or primary medium‐distal vessel occlusion (M3/M4, A2/A3, and P2/P3) as defined by Saver et al,^2^ treated with MT or MT+IVT were collected at 37 centers in North America, Asia, and Europe. Data were collected between September 2017 and July 2023. Data for this study were collected prospectively and reviewed retrospectively. The local neurointerventionalist reviewed all cases before sending their data to the MAD‐MT consortium. They determined the angiographic treatment success before the data was sent to the consortium, which was self‐reported by each center.

### Data Collection and Outcomes

Baseline clinical and demographic characteristics were recorded for patients and included sex (male or female), age, hypertension, hypercholesterolemia, diabetes, atrial fibrillation, and smoking status. Prestroke modified Rankin Scale score and occluded vessel were recorded. National Institutes of Health Stroke Scale (NIHSS) score was recorded at presentation. Baseline Alberta Stroke Program Early CT [Computed Tomography] Score was collected using noncontrast head computed tomography.^38^, ^42^

Other details of interest included antiplatelet and anticoagulation medication status, mothership versus drip‐and‐ship presentation, time from onset to puncture and recanalization, vital sign readings (blood pressure and heart rate), glycemic readings, anesthesia type (general, procedural sedation, or local), access site (femoral or radial), and imaging after MT (computed tomography, magnetic resonance, or none). Patients were divided into subgroups based on occlusion location at the first angiography run: medium proximal vessels: M2, A1, P1, and medium distal vessels: M3, A2, P2, and further.

### Outcome

The primary outcome of interest was excellent recanalization (mTICI 2c–3), secondary outcomes included favorable outcome (modified Rankin scale 0–2) at 90 days, excellent outcomes modified Rankin scale (0–1), and mortality at 90 days. Safety outcomes included intracerebral hemorrhage by any type and symptomatic intracerebral hemorrhage according to “The Heidelberg Bleeding Classification.”^43^

### Procedural and Technical Details

Treatment consisted of MT alone or MT+IVT. MT access site, either femoral or radial artery, and endovascular strategy (aspiration, stent retriever, combined or rescue techniques) were left to the individual operator's discretion. Similarly, the number of passes was left to the treating physician's discretion and institutional guidelines. The final mTICI scores was adjudicated as per each institution's protocol.^44^

### Statistical Analysis

Statistical analysis was conducted using R software (version 4.2.2).^45^ For descriptive statistics, categorical variables were presented as frequencies and percentages, while continuous variables were presented as medians with their interquartile ranges (IQRs). To explore the relationship between pretreatment and procedural factors and excellent recanalization, univariable logistic regression analysis was initially applied. Variables demonstrating a *P*‐value of <0.05 in this preliminary analysis were subsequently included in a multivariable logistic regression model. Backward stepwise selection was then applied with retention threshold with *P* value of 0.1. The results from the logistic regression analyses are reported as odds ratios (ORs) along with their 95% CIs. For the comparison of secondary and safety outcomes variables, categorical variables were analyzed using the χ^2^ test, and continuous variables were assessed using the Mann–Whitney *U* test. Adjusted effect estimates was calculated using logistic regression and linear regression for continues variables. All statistical tests were 2 sided, and a *P* value <0.05 was considered indicative of statistical significance.

## Results

### Baseline Characteristics

**Figure 1:**
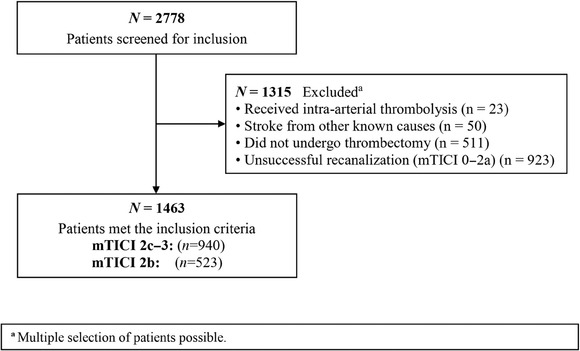
**Flow chart for patient selection**. mTICI indicates modified Thrombolysis in Cerebral Infarction.

A total of 2778 patients were screened for inclusion in the study. Of these, 1463 patients who achieved mTICI 2b–3 and met the inclusion criteria were included in the primary analysis (median age, 76 years [IQR, 65–83]; 762 females, 701 males). Among these patients, 523 achieved mTICI 2b recanalization, and 940 achieved mTICI 2c–3 recanalization (Figure 1). Hypertension was the most common comorbidity, present in 69% (361/523) of mTICI 2b patients and 73% (690/940) of mTICI 2c–3 patients (*P* = 0.12). Atrial fibrillation was observed in a similar proportion in both groups, with 36% (190/523) in mTICI 2b and 38% (353/940) in mTICI 2c–3 (*P* = 0.84) (Table 1).

**Table 1:** Predictors of Excellent Recanalization (mTICI 2c–3) in Patients With Proximal and Distal Medium Vessel Occlusions Stroke With Successful Recanalization (mTICI 2b–3)

	mTICI 2b	mTICI 2c–3	Univariable model		Multivariable model	
Variable	N=523	N=940	OR (95% CI)	*P* value	OR (95% CI)	*P* value
Pretreatment clinical parameters						
Age, y, median (IQR)	76 (65–83)	76 (63–82)	1.00 (1.00–1.01)	0.84		
Male drc, n (%)	240 (46)	461 (49)	1.12 (0.90–1.40)	0.31		
Hypercholesterolemia, n (%)	168 (33)	332 (36)	1.10 (0.87–1.39)	0.44		
Hypertension, n (%)	361 (69)	690 (73)	1.22 (0.95–1.55)	0.12		
Diabetes, n (%)	134 (26)	207 (22)	0.80 (0.62–1.03)	0.085		
Atrial fibrillation, n (%)	190 (36)	353 (38)	1.02 (0.82–1.29)	0.84		
Current smokers, n (%)	48 (9.2)	112 (12)	1.22 (0.85–1.78)	0.28		
Previous use of antiplatelet drugs, n (%)	136 (29)	275 (32)	1.14 (0.89–1.47)	0.3		
Previous use of anticoagulant drugs, n (%)	109 (24)	225 (27)	1.12 (0.85–1.47)	0.43		
Baseline mRS score, n (%)						
0	307 (62)	535 (60)	—			
1	67 (13)	149 (17)	1.34 (0.96–1.88)	0.088		
2	52 (10)	92 (10)	0.92 (0.63–1.36)	0.68		
3	50 (10)	89 (10.0)	1.01 (0.70–1.49)	0.95		
4	21 (4.2)	28 (3.1)	0.71 (0.39–1.30)	0.26		
Baseline NIHSS score, median (IQR)	12 (7–17)	10 (6–16)	0.98 (0.96–0.99)	0.006	0.99 (0.97–1.00)	0.087
Stroke cause, n (%)						
Large artery atherosclerosis	45 (8.6)	56 (6.0)	—		—	
Cardioembolic	171 (33)	346 (37)	1.63 (1.02–2.59)	0.041	1.67 (1.07–2.59)	0.023
Unknown pathogenesis despite workup	307 (59)	538 (57)	1.43 (0.91–2.24)	0.12	1.51 (0.98–2.33)	0.061
Pretreatment imaging parameters						
Site of initial occlusion, n (%)						
Medium (M2, P1, and A1)	453 (87)	746 (79)	—		—	
Distal (M3, P2, A2, and others)	70 (13)	194 (21)	1.49 (1.07–2.10)	0.019	1.58 (1.17–2.15)	0.003
ASPECTS, median (IQR)	9.00 (8.00–10.00)	9.00 (8.00–10.00)	1.09 (1.00–1.17)	0.039	96 (6.8)	40 (7.9)
Side, n (%)						
Right	275 (53)	475 (51)	—			
Left	243 (47)	458 (49)	1.12 (0.90–1.40)	0.32		
Mismatch ratio, median (IQR)	5 (2–18)	4 (3–22)	1.00 (1.00–1.00)	0.28		
Mismatch volume, median (IQR)	44 (32–79)	44 (24–76)	1.00 (0.99–1.01)	0.96		
Core volume (mL), median (IQR)	6 (0–25)	6 (0–19)	0.99 (0.99–1.00)	0.18		
T_max_ 10 volume (mL), median (IQR)	19 (5–36)	25 (6–46)	1.01 (1.00–1.02)	0.28		
T_max_ 8 volume (mL), median (IQR)	31 (16–58)	42 (15–69)	1.00 (0.99–1.01)	0.71		
T_max_ 6 volume (mL), median (IQR)	63 (43–92)	68 (33–93)	1.00 (0.99–1.00)	0.55		
T_max_ 4 volume (mL), median (IQR)	130 (92–176)	125 (80–174)	1.00 (1.0–1.00)	0.25		
Hypoperfusion intensity ratio, median (IQR)	0.12 (0.04–0.25)	0.17 (0.06–0.34)	3.38 (0.40–31.8)	0.27		
Procedural parameters						
Given IVT, n (%)	273 (52)	437 (46)	0.81 (0.65–1.01)	0.056	0.82 (0.66–1.01)	0.068
First‐line technique, n (%)						
Aspiration	88 (17)	195 (21)	—		—	
Both	350 (67)	630 (67)	0.80 (0.59–1.07)	0.13	0.80 (0.59–1.07)	0.14
Stent retriever	85 (16)	115 (12)	0.62 (0.42–0.92)	0.017	0.64 (0.43–0.93)	0.021
Mothership versus drip and ship, n (%)						
Drip and ship	226 (45)	401 (45)	—			
Mothership	276 (55)	500 (55)	0.97 (0.77–1.21)	0.77		
Onset to arterial puncture (minutes), median (IQR)	258 (178–409)	265 (175–425)	1.00 (1.00–1.00)	0.54		
Anesthesia, n (%)						
CS/LA	398 (76)	681 (73)	—			
GA	123 (24)	252 (27)	1.12 (0.87–1.45)	0.39		

ASPECTS indicates Alberta Stroke Program Early CT Score; CS/LA, conscious sedation/local anesthesia; CI, confidence interval; GA, general anesthesia; IQR, interquartile range; IVT, intravenous thrombolysis; mRS, modified Rankin scale; mTICI, modified Thrombolysis in Cerebral Infarction; NIHSS, National Institutes of Health Stroke Scale; and OR, odds ratio.

### Univariable Logistic Regression Model

In the univariable analysis, several factors were found to be associated with excellent recanalization (mTICI 2c–3). These included the site of initial occlusion, with distal occlusions (M3, P2, A2, and others) having higher odds of achieving mTICI 2c–3 (OR, 1.49; 95% CI, 1.07–2.10; *P* = 0.019). A lower baseline NIHSS score was also associated with a higher likelihood of excellent recanalization (OR, 0.98; 95% CI, 0.96–0.99; *P* = 0.006). Cardioembolic stroke pathogenesis was associated with higher odds of excellent recanalization (OR, 1.63; 95% CI, 1.02–2.59; *P* = 0.041), and the use of stent retrievers alone was associated with lower odds (OR, 0.62; 95% CI, 0.42–0.92; *P* = 0.017).

### Multivariable Logistic Regression Model

**Figure 2:**
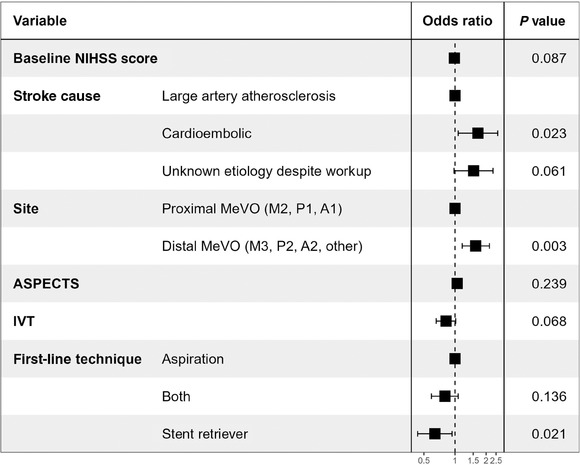
**Forest plot for predictors of excellent recanalization after mechanical thrombectomy in patients with MeVO stroke**. Forest plot displaying the ORs and *P* values for factors associated with excellent recanalization (mTICI 2c‐3) in acute ischemic stroke patients undergoing mechanical thrombectomy. The variables include baseline NIHSS score, IVT treatment, first‐line thrombectomy technique (aspiration, both, stent retriever), site of occlusion (medium, distal), stroke cause (large artery atherosclerosis, cardioembolic, unknown pathogenesis despite workup), ASPECTS, and prestroke mRS score. Black squares represent the ORs, and horizontal lines indicating the 95% CIs. The dashed vertical line represents an OR of 1. Variables with a *P* value <0.05 are considered statistically significant. ASPECTS indicates Alberta Stroke Program Early CT Score; IVT, intravenous thrombolysis; MeVO, medium vessel occlusion; mRS, modified Rankin Scale; mTICI, modified Thrombolysis in Cerebral Infarction; NIHSS, National Institutes of Health Stroke Scale; and OR, odds ratio.

In the multivariable logistic regression model (Figure 2), the site of initial occlusion was independently associated with excellent recanalization, with distal occlusions showing higher odds compared with proximal MeVO (adjusted OR [aOR], 1.58; 95% CI, 1.17–2.15; *P* = 0.003). Further, cardioembolic stroke cause was associated with a higher odds of excellent recanalization (aOR, 1.67; 95% CI, 1.07–2.59; *P* = 0.018) whereas the use of stent retrievers was associated with a lower odds of achieving excellent recanalization (aOR, 0.64; 95% CI, 0.43–0.93; *P* = 0.021). However, baseline NIHSS scores (aOR 0.99; 95% CI, 0.97–1.00; *P* = 0.087) and IVT administration (aOR 0.82; 95% CI, 0.66–1.01; *P* = 0.068) were not significantly associated with excellent recanalization (mTICI 2c‐3).

### Functional Outcomes

**Figure 3:**
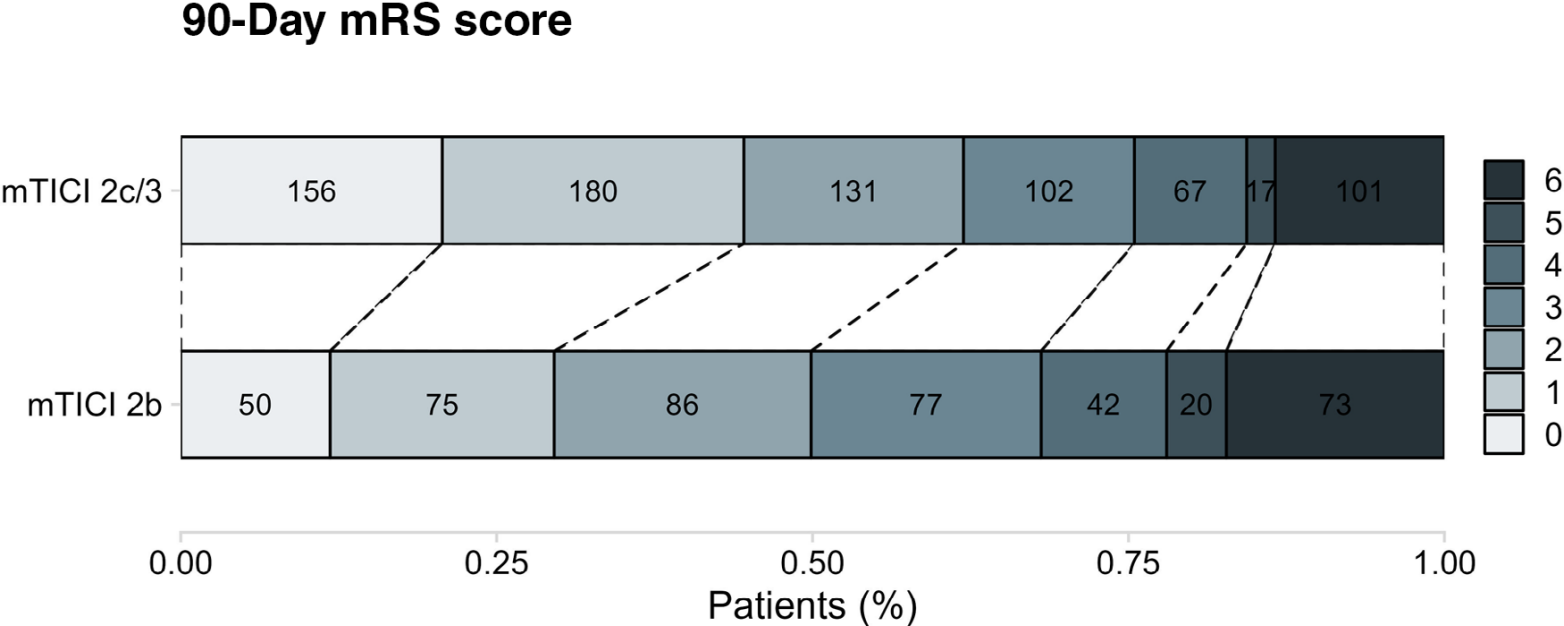
**Ninety‐day modified Rankin Scale score outcomes by recanalization group (mTICI b versus mTICI 2c–3)**. Stacked bar graph illustrating the distribution of 90‐day mRS scores in patients following mechanical thrombectomy, categorized into the mTICI b group, and the mTICI 2c/3 group. The numbers within the bars indicate the count of patients corresponding to each mRS score. The percentage of patients within each mRS category is plotted on the *x* axis. mTICI indicates modified Thrombolysis in Cerebral Infarction; and mRS, modified Rankin scale.

Day 1 median NIHSS score post‐MT was lower in the mTICI 2c–3 group (median 5, IQR 2–11) than in the mTICI 2b group (median 8, IQR 4–15, *P*<0.001) (Table 2). Similarly, the NIHSS shift showed a significant improvement in the mTICI 2c–3 group (median −3, IQR −8 to 0) compared with the mTICI 2b group (median −2, IQR −6 to 2, *P*<0.001). In terms of 90‐day outcomes, a higher percentage of patients in the mTICI 2c–3 group achieved modified Rankin scale score of 0–1 (45% versus 30% in the mTICI 2b group, *P*<0.001) and 0–2 (62% versus 50% in the mTICI 2b group, *P*<0.001). The 90‐day mortality rate was lower in the mTICI 2c–3 group (13%) compared with the mTICI 2b group (17%), but this difference was not statistically significant (*P* = 0.088). (Figure 3). These outcomes did not change after adjustment (Table 3).

**Table 2:** Safety and Functional Outcomes

		mTICI 2b	mTICI 2c–3	
Variable	Overall, N=1463	N=523	N=940	*P*‐**value**
Total number of passes, median (IQR)	2.00 (1.00 to 2.00)	2.00 (1.00 to 3.00)	1.00 (1.00 to 2.00)	<0.001***
Day 1 NIHSS score, median (IQR)	6 (2 to 13)	8 (4 to 15)	5 (2 to 11)	<0.001***
NIHSS shift, median (IQR)	−3 (−7 to 1)	−2 (−6 to 2)	−3 (−8 to 0)	<0.001***
90‐d mRS score 0–1, n (%)	461 (39)	125 (30)	336 (45)	<0.001***
90‐d mRS score 0–2, n (%)	678 (58)	211 (50)	467 (62)	<0.001***
90‐d mortality, n (%)	174 (15)	73 (17)	101 (13)	0.088
sICH, n (%)	96 (6.8)	40 (7.9)	56 (6.2)	0.25
Intracranial hemorrhage (any type), n (%)	479 (34)	177 (35)	302 (34)	0.59
Intracranial hemorrhage (by type), n (%)				0.85
HI1	278 (60)	104 (60)	174 (59)	
HI2	26 (5.6)	9 (5.2)	17 (5.8)	
PH1	30 (6.5)	11 (6.4)	19 (6.5)	
PH2	21 (4.5)	10 (5.8)	11 (3.8)	
SAH	110 (24)	38 (22)	72 (25)	
Embolization in new territories, n (%)	42 (2.9)	27 (5.2)	15 (1.6)	<0.001***
Perforation, n (%)	37 (2.5)	25 (4.8)	12 (1.3)	<0.001***
Artery dissection, n (%)	14 (1.0)	6 (1.2)	8 (0.9)	0.78

IQR, interquartile range; mRS, modified Rankin Scale; mTICI, modified Thrombolysis in Cerebral Infarction NIHSS, National Institutes of Health Stroke Scale; OR, odds ratio; SAH, subarachnoid hemorrhage; sICH; and symptomatic intracerebral hemorrhage.

**Table 3:** Unadjusted Effect Estimate and Adjusted Effect Estimate Using Logistic and Linear Regression Models

	Unadjusted effect estimate		Adjusted effect estimate^†^	
Outcome	OR/Beta (95% CI)^*^	*P*‐**value**	OR/Beta (95% CI)^*^	*P*‐**value**
90‐d mRS score 0–1	1.92 (1.49 to 2.47)	<0.001	1.89 (1.38 to 2.60)	<0.001
90‐d mRS score 0–2	1.63 (1.29 to 2.08)	<0.001	1.83 (1.34 to 2.50)	<0.001
90‐d mortality	0.74 (0.54 to 1.03)	0.074	0.71 (0.49 to 1.04)	0.078
ICH (any type)	0.93 (0.74 to 1.17)	0.55	0.92 (0.72 to 1.19)	0.52
HI1	0.92 (0.70 to 1.21)	0.55	0.92 (0.68 to 1.24)	0.58
HI2	1.06 (0.48 to 2.50)	0.89	1.85 (0.73 to 5.35)	0.22
PH1	0.97 (0.46 to 2.12)	0.93	0.79 (0.36 to 1.79)	0.56
PH2	0.61 (0.26 to 1.48)	0.26	0.74 (0.26 to 2.20)	0.58
SAH	1.07 (0.71 to 1.62)	0.76	0.85 (0.53 to 1.37)	0.49
24 h NIHSS shift^‡^	−1.7 (−2.6 to −0.84)	<0.001	−1.9 (−2.8 to −0.93)	<0.001
Day 1 NIHSS score	−2.8 (−3.8 to −1.9)	<0.001	−2.4 (−3.3 to −1.5)	<0.001

ASPECTS indicates Alberta Stroke Program Early CT Score; ICH, intracerebral hemorrhage; IQR, interquartile range;mRS, modified Rankin scale; NIHSS, National Institutes of Health Stroke Scale; OR, odds ratio; and SAH, subarachnoid hemorrhage;.

* **Effect estimates are provided as Beta with 95% CIs for 24‐hour NIHSS shift and day 1 NIHSS score**.

^†^ All adjusted estimates were adjusted for sex, age, high blood pressure, high cholesterol, diabetes, atrial fibrillation, smoking, mRS score before stroke, baseline NIHSS score, ASPECTS, and occlusion site.

^‡^ **Admission NIHSS was not included in the adjustment**.

### Safety Outcomes

Regarding safety outcomes, symptomatic intracerebral hemorrhage occurred in 7.9% of the mTICI 2b group and 6.2% of the mTICI 2c–3 group (*P* = 0.25). Intracerebral hemorrhage of any type was observed in 35% of the mTICI 2b group and 34% of the mTICI 2c–3 group (*P* = 0.59). The types of intracranial hemorrhage did not differ significantly between the groups (*P* = 0.85). Embolization in new territories and perforation were significantly less frequent in the mTICI 2c–3 group (embolization: 1.6% versus 5.2% in mTICI 2b, *P*<0.001; perforation: 1.3% versus 4.8% in mTICI 2b, *P*<0.001). The incidence of artery dissection was similar between the 2 groups (1.2% in mTICI 2b versus 0.9% in mTICI 2c–3, *P* = 0.78). Safety outcomes maintained nonsignificance after adjustment (Table 3).

## Discussion

In this multicenter, multinational study, we have elucidated several factors that are independently associated with achieving excellent recanalization (mTICI 2c–3) in patients with MeVO stroke who achieved successful recanalization (mTICI 2b–3). One of the main findings is the observation that individuals with distal occlusions exhibited higher odds for achieving excellent recanalization than proximal MeVO. Additionally, a cardioembolic stroke pathogenesis emerged as a significant independent variable, particularly when compared with strokes stemming from atherosclerotic rupture. Intriguingly, the use of stent retrievers alone was found to be inversely associated with the achievement of excellent recanalization.

Better clinical outcomes were observed in patients who achieved mTICI 2c–3 recanalization compared with mTICI 2b recanalization group, characterized by lower initial NIHSS scores, and more pronounced improvements in NIHSS scores. These patients also exhibited higher rates of favorable outcomes at 90 days. However, the study found no significant difference in the 90‐day mortality rates between the different recanalization groups. In terms of safety, the incidence of symptomatic intracerebral hemorrhage and other types of intracerebral hemorrhage was comparable across the groups, although the probability of embolization in new territories was greater in patients with mTICI 2b recanalization group.

Our study's observation that patients with cardioembolic strokes were more likely to achieve excellent recanalization (mTICI 2c–3) aligns with existing literature. Previous studies have indicated that strokes due to atherosclerosis are often associated with less successful outcomes in thrombectomy procedures.^46^, ^47^, ^48^, ^49^ This phenomenon may partly attributed to distinct thrombus characteristics of the underlying stroke pathogenesis, which in turn may affect the success rates of MT. For example, successful revascularization in cases of underlying atherosclerosis may be more challenging due to possible residual focal stenosis, in situ thrombosis, and a higher propensity for reocclusion.^50^, ^51^, ^52^, ^53^, ^54^, ^55^, ^56^, ^57^

The higher odds of excellent recanalization in distal occlusions can be attributed to thrombus composition in these smaller vessels. Thrombi in distal occlusions often differ from those in proximal occlusions, potentially affecting the efficacy of MT. Additionally, thrombi in smaller vessels tend to be more susceptible to mechanical disruption. This susceptibility could contribute to the increased rates of successful recanalization observed in distal occlusions.^58^, ^59^, ^60^, ^61^ This supports the notion of the feasibility of endovascular therapy for distal intracranial occlusions, though the extent of its beneficial effects warrants further exploration.^10^, ^38^, ^62^, ^63^, ^64^, ^65^, ^66^ Conversely, the fact that distal occlusions often result in better recanalization outcomes than proximal ones defies traditional expectations, given the technical challenges in accessing and manipulating distal occlusions during MT. This paradox invites further speculation on whether the difficulty in distinguishing between different grades of reperfusion (eg, mTICI) in smaller, more distal occlusions might explain these unexpected results.

In our analysis, the aspiration‐only technique emerged as an independent predictor of excellent recanalization. This association might reflect the intricacies involved in the thrombectomy procedure, particularly the influence of clot characteristics on the success of aspiration‐only methods.^27^, ^67^, ^68^, ^69^ Conversely, the need for stent retriever, often indicative of more complex occlusions, may explain the robust correlation between the choice of MT technique and the achievement of excellent recanalization.^70^, ^71^, ^72^ However, given the inherent differences in procedural complexity between aspiration‐only and stent retriever technique, these findings should be interpreted with caution. The retrospective nature of our study further necessitates a prospective approach in drawing definitive conclusions from this observation.

Our study has multiple strengths, including large‐scale, multinational, multicenter, and real‐world data, thereby improving generalizability. However, our research was limited by its retrospective nature and inherent selection bias, which could influence the study's outcomes and generalizability. Additionally, the findings should be interpreted with caution due to the heterogeneity of MeVO, which includes a range of anatomical locations and vessel sizes. This heterogeneity can significantly affect both therapeutic decisions and outcomes and may confound the evaluation of other factors associated with excellent recanalization (mTICI 2c‐3).

Despite the widespread use of the mTICI score, it has notable limitations, particularly for distal occlusions. The mTICI score was originally designed for larger vessel occlusions and may not accurately capture the nuances of perfusion in smaller, more distal vessels. In distal occlusions, distinguishing between different grades of reperfusion (mTICI 2b versus mTICI 2c–3) can be challenging due to the smaller vessel caliber, which diminishes the precision of the mTICI score in these cases.^73^

Moreover, our data do not categorize M2 occlusions into codominant or nondominant segments, a factor that could potentially influence outcomes and procedural success. Future studies should explore the validity of mTICI in distal MeVO and consider investigating alternative or supplementary scoring systems that better capture the reperfusion status in distal vessel occlusions. Additionally, prospective studies are warranted to validate our results and further investigate other factors associated with excellent recanalization in patients with MeVO stroke.

## Conclusion

Our comprehensive study, encompassing a large, multinational patient cohort, has highlighted key factors associated with excellent recanalization (mTICI 2c–3) among patients with MeVO stroke who achieved successful recanalization (mTICI 2b–3) using MT. Patients with distal occlusions were more likely to achieve excellent recanalization compared with those with proximal MeVO, suggesting a wider applicability of MT in MeVO cases. The study also revealed that cardioembolic stroke pathogenesis was associated with a higher likelihood of excellent recanalization. Clinically, patients achieving mTICI 2c–3 recanalization exhibited better outcomes, including lower initial NIHSS scores and higher rates of favorable outcomes at 90 days. Further validation through prospective studies is necessary to confirm the validity of our results.

## Sources of Funding

No funds, grants, or other support were received for conducting this study or to assist with the preparation of this manuscript.

## Disclosures

Dr Regenhardt serves on a Data and Safety Monitoring Board for a trial sponsored by Rapid Medical, serves as site principal investigator for studies sponsored by Penumbra and Microvention, and receives stroke research grant funding from the National Institutes of Health, Society of Vascular and Interventional Neurology, and Heitman Stroke Foundation. Dr Guenego reports consultancy for Rapid Medical and Phenox, not directly related to the present work. Dr Clarençon reports conflicts of interest with Medtronic, Balt Extrusion (consultant), ClinSearch (core lab), Penumbra, Stryker (payment for reading), and Artedrone (Board); all not directly related to the present work. Dr Henninger received support from W81XWH‐19‐PRARP‐RPA form the Congressionally Directed Medical Research Programs/Department of Defense, NS131756 and U24NS113844 from the National Institute of Neurological Disorders and Stroke, and NR020231 from the National Institute of Nursing Researc and received compensation from Myrobalan, Inc. and General Dynamics during the conduct of this study unrelated to this work. Dr Liebeskind is consultant as Imaging Core Lab to Cerenovus, Genentech, Medtronic, Stryker, Rapid Medical. Dr Yeo reports advisory work for AstraZeneca, substantial support from National Medical Research Council Singapore, and is a medical advisor for See‐mode, Cortiro, and Sunbird Bio, with equity in Ceroflo. All unrelated to the present work. Dr Griessenauer reports a proctoring agreement with Medtronic and research funding by Penumbra. Dr Marnat reports conflicts of interest with Microvention Europe, Stryker Neurovascular, Balt (consulting), Medtronic, Johnson & Johnson and Phenox (paid lectures), all not directly related to the present work. Dr Puri is a consultant for Medtronic Neurovascular, Stryker NeurovascularBalt, Q'Apel Medical, Cerenovus, Microvention, Imperative Care, Agile, Merit, CereVasc and Arsenal Medical, he received research grants from National Institutes of Health, Microvention, Cerenovus, Medtronic Neurovascular, and Stryker Neurovascular, and holds stocks in InNeuroCo, Agile, Perfuze, Galaxy, and NTI. Dr Tjoumakaris is a consultant for Medtronic and Microvention (funds paid to institution, not personally). Dr Jabbour is a consultant for Medtronic, Microvention, and Cerus. Vitor Mendes Pereira, David S Liebeskind, Thanh N. Nguyen and James E. Siegler serve on the Editorial Board of S:VIN. Editorial Board Members are not involved in the handling or final disposition of submissions. Sunil A. Sheth is an Associate Editor for S:VIN and was not involved in the handling or final disposition of this article. Disclosures provided by Sunil A. Sheth in compliance with American Heart Association's annual Journal Editor Disclosure Questionnaire are available at https://www.ahajournals.org/editor-coi-disclosures.
